# Pennsylvania State Livestock Sanitary Board

**Published:** 1901-02

**Authors:** Leonard Pearson

**Affiliations:** State Veterinarian; Philadelphia


PENNSYLVANIA STATE LIVESTOCK SANITARY BOARD.

Dear Doctor: The replies that were received to the circular letter that was mailed to you in October indicate that there is a place for a communication of this character. Still some of those from whom replies were expected and desired did not answer, and if replies to subsequent inquiries are not received their silence will be interpreted to mean that they do not care to receive these circulars.

1. *Methods of Computing the Prevalence of Infectious Diseases among Animals.* One of the questions that was asked in the last circular letter was in regard to the prevalence of tuberculosis among animals in 1896 and also in 1900. This question seems to have caused some confusion, and for this reason I offer the following suggestion in regard to computing the prevalence of this and other diseases.

A basis of a computation or an estimate of this kind must be as broad and as accurate as possible. In estimating the prevalence of tuberculosis in a district one should use as a basis the herds with whose condition he is quite familiar. For example, twenty-five herds, representing the average conditions of the locality, and with which the veterinarian is familiar, may be taken as a standard. Being acquainted with these herds, the prevalence of tuberculosis in them can be estimated with a fair degree of accuracy. Some of them, and perhaps all of them, have been tested with tuberculin. If these are average herds and represent average conditions, conclusions drawn from a study of them may be applied generally to the herds in the district they represent.

In estimating the prevalence of other less common diseases information in regard to them must be derived from all available sources, but chiefly from knowledge gained in practice, from other veterinarians, from physicians, and from stockmen.

2. *In Regard to Appraising Cattle that are Condemned as Tubercular.* The payments made by the State for tubercular cattle are paid for cattle that it is necessary to condemn and destroy in order to restrict the spread of disease. These payments are made because under some conditions it is in the interests of the public that property of an individual shall be condemned and destroyed for the protection of the many. It should be borne in mind that the State does not appropriate money for the relief of an individual who suffers loss from the prevalence of disease, nor does it appropriate money for the relief of an individual who suffers loss from fire or floods or storm or drouth.

Tubercular cattle are paid for not to reimburse the individual for his loss, but to pay for property that has some value when it is necessary to take it for the protection of the public. It seems well to mention this principle and to endeavor to impress it upon those who are concerned with the enforcement of the law governing the diseases of animals. If this principle were borne in mind one would not ask, Can Mr. So and So, whose cow died of tuberculosis three weeks ago, receive compensation from the State? Of course, it would be contrary to the principle above stated and to the law to make any allowance in such a case. To do so would be to transform the work of the Livestock Sanitary Board into a free livestock insurance operation. There is no more reason why the State should pay for a cow that has died with tuberculosis than it should pay for a cow that has fallen into a well, but there is reason why the State should purchase and destroy living tubercular cows that are capable of spreading disease to cattle belonging to others.

3. *The Inspection of Dairy Cows and Cattle for Breeding Purposes that are Brought into Pennsylvania from Other States.* This inspection, which commenced on January 1, 1898, is carried out for a twofold purpose: (*a*) To prevent the importation of tubercular cattle into Pennsylvania and (*b*) to make it possible for those who wish to buy inspected cattle to do so without undue difficulty. This law has a further educational advantage, in that it has a tendency to teach dealers to buy cattle known to be sound and to teach purchasers the advantage of buying cattle that are tested. It is believed that the effect of this law is to very greatly restrict the spread of tuberculosis in this State.

In Massachusetts the enforcement of a similar law has been rendered difficult by the dishonesty of some of the examining veterinarians. The inspections that have been made of cattle coming into Pennsylvania have been carefully supervised, and it is gratifying to know that there is but little evidence of fraudulent inspections. There are, however, a few instances wherein veterinarians have rendered false reports. When it is discovered that this had occurred no further reports will be received from the dishonest individual, and the shippers who have been employing him are notified to this effect.

It has recently been necessary to drop from the list of approved veterinarians the names of three practitioners in Pennsylvania. Two of these men reported tuberculin tests that they had never made. They saw the cattle that they reported upon, examined them physically, and then wrote out reports indicating that complete tuberculin tests had been carried out. The third practitioner took temperatures and injected tuberculin in a proper way, and then did not return to see the cattle until the second day, and no temperatures had been taken in the interval. His report indicated that he had made a proper test.

It is astonishing that men practising as veterinarians would lend themselves to such dishonorable and despicable practices. Such work as this has a tendency to throw the entire interstate examination and the tuberculin test into disrepute. Furthermore, it casts a serious reflection upon the veterinary profession. It is not fair to judge the interstate examination, or the tuberculin test, or the veterinary profession by these exceptional occurrences, but there is a tendency to judge by just these things.

Any veterinarian who is shown to be guilty of such work should not only be dropped from the list of approved veterinarians in the office of the State Livestock Sanitary Board and be refused the privilege of doing work for the State, but he should also be ostracised and condemned by every right thinking veterinarian and honest citizen.

As I have said, these derelictions are rare, and the examination in practically all cases is made as the law directs; so the law cannot validly be objected to on the ground that the inspections are not honestly made. There is an objection to it by some dealers on the ground of delay and expense, but the delay and expense are not material and cannot be compared with the loss to the farmer who unwittingly buys tubercular cattle. It is my experience that the dairymen in the eastern part of the State want this law for the protection that it affords them, while the stock raisers in the central and western parts of the State want this law because it helps their market.

At the last session of the Legislature a member of the House, who was a dealer in cattle, introduced an act to repeal the law under consideration. The proposition was negatived by the Committee on Agriculture. It is possible that a similar proposition may come up at this session. In this case it is important that the voice of those who believe that the law is a good one shall be heard.

4. *The Special Course for Veterinarians to be given in Philadelphia at the Time of the State Meeting.* For one week preceding the meeting last year of the State Veterinary Medical Association a special course of instruction was given to the veterinarians who cared to avail themselves of it. This instruction was in the nature of post-graduate work in clinical surgery, infectious diseases, meat-, milk-, and dairy-inspection, bacteriology, zoötechnics, and special discussions on the work of the State Livestock Sanitary Board. At a dinner held during the course it was voted that it would be desirable to repeat the work the following year. In accordance with this unanimous request of the veterinarians in attendance last year it has been arranged to provide a course this year, beginning on Monday, March 4th, and ending on Saturday, March 9th. Two days of this time will be occupied by the meeting of the State Veterinary Society; the other four days will be devoted to clinical surgery, in which new operations will be demonstrated; to contagious diseases, in which recent knowledge in this connection will be discussed; bacteriology in its practical application; dairy-inspection, milk-inspection, with demonstrations; feeding animals and compounding rations for special purposes; the construction of stables, breeding problems, the work of the Livestock Sanitary Board, etc. Morning, afternoon, and evening sessions will be held and the time will be well filled.

5. *Forage Poisoning in Cows.* Since the reference to forage poisoning in my last circular a great quantity of valuable information on this subject has been received. Many reports of forage poisoning of horses have been received and also special reports of what is believed to be forage poisoning of cattle. In this condition there is difficulty in swallowing, muscular tremors and weakness, sometimes inability to stand, and death within a few days.

The cause is usually attributed to very mouldy corn fodder or ensilage. The treatment that is most highly esteemed is a purge, a few small doses of creolin and nux vomica given by the mouth, or strychnine hypodermatically. I should be very glad to know to what extent this disease has occurred in various parts of the State. There are many sudden deaths among cattle that are due to causes that it is difficult to fathom. Perhaps this is an explanation for some of them.

6. A new edition is being prepared and is now in press of the article on "Tuberculosis" that appeared in the *Report of the Department of Agriculture* for 1899, with additions that have brought this article down to date and amplified it considerably. It is being published as a separate report of about 275 pages. A copy will be sent to you when it is issued.

Yours truly,

LEONARD PEARSON,

PHILADELPHIA, February 4, 1901. State Veterinarian.

